# The life cycle-dependent transcriptional profile of the obligate intracellular amoeba symbiont *Amoebophilus asiaticus*

**DOI:** 10.1093/femsec/fiac001

**Published:** 2022-01-06

**Authors:** E Selberherr, T Penz, L König, B Conrady, A Siegl, M Horn, S Schmitz-Esser

**Affiliations:** Unit of Food Microbiology, Institute of Food Safety, Food Technology and Veterinary Public Health, Department for Farm Animals and Veterinary Public Health, University of Veterinary Medicine Vienna, 1210 Vienna, Austria; Centre for Microbiology and Environmental Systems Science, University of Vienna, 1030 Vienna, Austria; Centre for Microbiology and Environmental Systems Science, University of Vienna, 1030 Vienna, Austria; Department of Veterinary and Animal Science, University of Copenhagen, 1870, Denmark; Centre for Microbiology and Environmental Systems Science, University of Vienna, 1030 Vienna, Austria; Centre for Microbiology and Environmental Systems Science, University of Vienna, 1030 Vienna, Austria; Department of Animal Science, Iowa State University, Ames, IA, 50011, USA

**Keywords:** *Amoebophilus asiaticus*, amoeba, symbiont, transcriptome, type 6 secretion system, eukaryotic-like proteins

## Abstract

Free-living amoebae often harbor obligate intracellular bacterial symbionts. *Amoebophilus (A.) asiaticus* is a representative of a lineage of amoeba symbionts in the phylum *Bacteroidota*. Here, we analyse the transcriptome of *A. asiaticus* strain 5a2 at four time points during its infection cycle and replication within the *Acanthamoeba* host using RNA sequencing. Our results reveal a dynamic transcriptional landscape throughout different *A. asiaticus* life cycle stages. Many intracellular bacteria and pathogens utilize eukaryotic-like proteins (ELPs) for host cell interaction and the *A. asiaticus* 5a2 genome shows a particularly high abundance of ELPs. We show the expression of all genes encoding ELPs and found many ELPs to be differentially expressed. At the replicative stage of *A. asiaticus*, ankyrin repeat proteins and tetratricopeptide/Sel1-like repeat proteins were upregulated. At the later time points, high expression levels of a type 6 secretion system that likely prepares for a new infection cycle after lysing its host, were found. This study reveals comprehensive insights into the intracellular lifestyle of *A. asiaticus* and highlights candidate genes for host cell interaction. The results from this study have implications for other intracellular bacteria such as other amoeba-associated bacteria and the arthropod symbionts *Cardinium* forming the sister lineage of *A. asiaticus*.

## Teaser

This study reveals the first comprehensive insights into the intracellular lifestyle of the obligate intracellular amoeba symbiont *A. asiaticus* and highlights candidate genes for host cell interaction.

## Introduction

Free-living amoebae are protozoa that are ubiquitous in water and soil and are important predators in these environments by feeding on other microorganisms, mainly bacteria, and digesting their prey through phagocytosis (Samba-Louaka *et al*. 2019). Thus, free-living amoebae play an important role in the microbial ecology of these environments. Over the last few decades, the interest in free-living amoebae has increased, based on the discovery that several human pathogens can resist phagocytosis and replicate in these protists, such as *Legionella* or *Mycobacterium* species (Greub and Raoult 2004). In addition, amoebae likely play a role in the evolution of amoeba-associated microorganisms, including several human pathogens. Several lines of evidence exist suggesting that amoebae and other protozoa contribute to the adaption of bacteria to new intracellular environments in higher eukaryotic organisms such as insects and mammals (Penz, Horn and Schmitz-Esser 2010; Toft and Andersson 2010). Therefore, it has been proposed that amoebae can be ‘training grounds’ for intracellular pathogenic bacteria (Molmeret *et al*. 2005).

Prior research has shown that, in addition to human pathogens, free-living amoebae can harbor a variety of obligate intracellular bacterial symbionts, affiliating with several bacterial phyla (Horn and Wagner 2004; Samba-Louaka *et al*. 2019; Brock *et al*. 2020; Haselkorn *et al*. 2021). *Amoebophilus asiaticus* is an obligate intracellular amoeba symbiont that was first discovered in an amoeba isolated from lake sediment samples (Horn *et al*. 2001; Schmitz-Esser *et al*. 2008). It belongs to a lineage of endosymbionts and coral-associated sequences within the phylum of *Bacteroidota* named *Amoebophilaceae*, with the arthropod symbiont *Cardinium hertigii* representing a sister lineage of *A. asiaticus* (Santos-Garcia *et al*. 2014). In addition to the identification of different *A. asiaticus* strains as amoeba symbionts (Xuan *et al*. 2007; Schmitz-Esser *et al*. 2008; Choi *et al*. 2009; Lagkouvardos, Shen and Horn 2014; Haselkorn *et al*. 2021), *A. asiaticus* sequences have also been identified in soil metagenomes (Alteio *et al*. 2020), coral microbiomes (Sunagawa, Woodley and Medina 2010; Apprill, Weber and Santoro 2016; Huggett and Apprill 2019) and in a digital dermatitis metagenome from cattle (Zinicola *et al*. 2015a,b). *Amoebophilus asiaticus* possesses a biphasic life cycle consisting of an extracellular coccoid infectious stage and an intracellular rod-shaped replicating stage (Penz, Horn and Schmitz-Esser 2010; Bock *et al*. 2017). Outside the amoeba host, A. *asiaticus* cannot replicate under environmental conditions (Schmitz-Esser *et al*. 2010). After initial attachment to the amoebal surface and subsequent phagocytosis, *A. asiaticus* escapes from the phagosome and replicates until the cytoplasm of amoeba trophozoites and cysts is nearly completely filled with *A. asiaticus*. Finally, *A. asiaticus* lyses its host to start a new infectious cycle (Horn *et al*. 2001; Schmitz-Esser *et al*. 2008; Bock *et al*. 2017). These studies characterizing the *A. asiaticus* life cycle were also the basis for selecting time points for RNA extraction in this experiment using fluorescence *in situ* hybridization (FISH) experiments analyzing the *A. asiaticus* life cycle to identify characteristic life cycle stages: extracellular and early intracellular (up to 12 hours post-infection (h p.i.)), intracellular replicative stage (12 to 72 h p.i.) and late and extracellular stage (144 h p.i. to extracellular; Horn *et al*. 2001; Schmitz-Esser *et al*. 2008; Penz 2014; Bock *et al*. 2017).

In its host, *A. asiaticus* is not surrounded by a host-derived membrane, allowing for direct uptake of essential compounds like amino acids or nucleotides from the host cell cytoplasm (Schmitz-Esser *et al*. 2008; Penz, Horn and Schmitz-Esser 2010). It is supposed that substrates from the *Acanthamoeba* host are the main nutrient source for *A. asiaticus*. Compared with obligate intracellular insect symbionts such as *Buchnera*, which are characterized by extremely reduced genome sizes, *A. asiaticus* has an only moderately reduced genome of 1.9 Mbp but its biosynthetic capability is highly limited (Schmitz-Esser *et al*. 2010). For example, a crucial enzyme of glycolysis, the phosphofructokinase, as well as the tricarboxylic acid cycle and pathways for the biosynthesis of nucleotides, cofactors and amino acids, are missing (Schmitz-Esser *et al*. 2010). Instead, a variety of predicted transporter genes (*n* = 82), including a putative ATP/ADP translocase, an S-adenosylmethionine (SAM) carrier (Alteio *et al*. 2020) and oligopeptide/proton symporters are encoded in the *A. asiaticus* genome.

Another remarkable feature of *A. asiaticus* and other amoeba symbionts are highly abundant genes encoding eukaryotic-like proteins (ELPs) including ankyrin (ANK)-, leucine-rich- and tetratricopeptide/Sel1-like (TPR/SEL1)-repeats, and proteins harboring domains predicted for interaction with the host ubiquitin system (Schmitz-Esser *et al*. 2010; Domman *et al*. 2014; Frank 2019; Mondino, Schmidt and Buchrieser 2020). These ELPs are thus likely involved in the interaction between *A. asiaticus* and its amoeba host (Schmitz-Esser *et al*. 2010). In general, intracellular symbiotic bacteria are broadly enriched with proteins harboring ANK repeats (Jernigan and Bordenstein 2014). ANK repeats of amoeba-associated bacteria, such as AnkB from *Legionella pneumophila*, have been shown to be critical for host cell interaction with humans and in amoebae (Lomma *et al*. 2010; Price *et al*. 2011). ANK repeats are, therefore, considered to be part of a system ensuring bacterial survival in eukaryotic host cells (Jernigan and Bordenstein 2014). Similarly, leucine-rich- and TPR/SEL1-repeats are involved in protein–protein interactions (Mittl and Schneider-Brachert 2007; Bella *et al*. 2008) and mediate bacteria–host interactions in different bacterial pathogens, including the amoeba-associated *L. pneumophila* (Bandyopadhyay *et al*. 2012; Cerveny *et al*. 2013).

The characterization of obligate intracellular endosymbionts like *A. asiaticus* is dependent on molecular methods because cultivation outside their host is currently not possible. In this study, we describe the course of the infection and characterize the transcriptome of *A. asiaticus* 5a2 at four key time points during its biphasic life cycle. Sequencing entire transcriptomes (RNA-seq) of host-associated bacteria has facilitated comprehensive insights into the life cycle of *A. asiaticus* 5a2, and the identification of factors and themes it has in common with the related insect symbiont *C. hertigii* whose transcriptome has been sequenced recently (Mann *et al*. 2017). Focusing on the role of predicted host-interaction genes, we aimed at understanding strategies accompanying the transcriptional transitions during the *A. asiaticus* life cycle to increase our knowledge on the adaptation of these bacteria to their intracellular niche.

## Methods

### Cell culture and infection experiment

*Acanthamoeba* sp. 5a2 is naturally infected with *A. asiaticus* strain 5a2 (ATCC number PRA-228) and was isolated from a lake sediment (Schmitz-Esser *et al*. 2008). Infected and uninfected *Acanthamoeba* sp. 5a2, were maintained as adherent cultures in cell culture flasks (25 cm^2^, Nalgene Nunc International, Rochester, NY). Flasks contained 10 mL trypticase soy broth (30 g/L) with yeast extract (10 g/L) with a pH of 7.3. Cell cultures were incubated at 27°C. They were passaged at confluency every 5–10 days (1:10 dilution).

For the infection experiment, symbiont-free *Acanthamoeba* 5a2 were harvested 3 days before infection and inoculated in one 500 cm^2^ cell culture flask (Nalgene Nunc International) per time point and replicate, and incubated at 27°C until infection. For this, symbiont-free *Acanthamoeba* 5a2 were infected with extracellular *A. asiaticus* 5a2 that were freshly released from *Acanthamoeba* sp. 5a2 cells. Of note, we observed that infection experiments were only successful using naturally and freshly released *A. asiaticus* cells, suggesting that only freshly released, extracellular *A. asiaticus* cells are capable of infecting new amoeba host cells. Symbiont-free amoebae were infected with a multiplicity of infection (MOI) of 500. The high MOI was required to obtain sufficiently high cell densities for the time points early after infection. Infected *Acanthamoeba* cultures were washed with trypticase soy broth to remove bacteria that were not taken up by its *Acanthamoeba* host. At different time points post-infection, *Acanthamoeba* sp. 5a2 infected with *A. asiaticus* were detached from the culture flask surface by shaking and harvested by centrifugation at 7000 rpm (3 min, 27°C). *Acanthamoeba* sp. 5a2 were then lysed in trypticase soy broth using a Dounce tissue grinder (Wheaton, Millville, NJ). The homogenized cell suspension was passed through a 5 µm pore-size cellulose filter (Sartorius, Göttingen, Germany) to separate bacterial cells from the *Acanthamoeba* host lysate. *Amoebophilus asiaticus* cells were stained with DAPI (4',6-diamidino-2-phenylindole) on a 0.22 μm polycarbonate membrane filter (EMD Millipore, Billerica, MA) and the number of *A. asiaticus* cells was monitored by counting with an epifluorescence microscope (Axioplan 2 imaging; Carl Zeiss, Oberkochen, Germany). Extracellular *A. asiaticus* were obtained by centrifugation (8000 rpm, 5 min, 27°C) of freshly released *A. asiaticus* stages which were harvested from well-grown infected *Acanthamoeba* cultures. To remove *Acanthamoeba* host debris, bacterial suspensions were passed through a 5 µm pore-size cellulose filter (Sartorius). All harvested bacteria were immediately processed.

### RNA isolation and sequencing

RNA from amoebae infected with freshly prepared cell suspensions of *A. asiaticus* 5a2 was isolated from one 500 cm^2^ cell culture flask per time point and replicated at the following time points: 12, 72, 144 h p.i. and from freshly released extracellular *A. asiaticus* (EC) after harvesting as described above. For each time point, three replicate cultures were included. Thus, we performed RNA extraction from a total of 12 cell culture flasks. These time points were selected based on FISH experiments analyzing the *A. asiaticus* life cycle to identify characteristic life cycle stages, and also were confirmed in previous studies (Penz 2014; Bock *et al*. 2017); representative FISH images of the *A. asiaticus* life cycle are shown in (Figure S1, Supporting Information). Cell pellets were immediately resuspended in 750 μL TRIzol (Invitrogen Life Technologies, Vienna, Austria). Cells were disrupted by beat-beating for 30 s at a speed of 4.5 with lysing matrix A tubes and a BIO101/Savant FastPrep FP120 instrument (MP Biomedicals, Santa Ana, CA). RNA was extracted according to the manufacturer's recommendations (TRIzol, Invitrogen Life Technologies), and remaining DNA was removed using the TURBO DNA-free Kit (Ambion, Vienna, Austria) with 10 µg nucleic acids digested in two subsequent steps with 3 U of DNase each. RNA was precipitated with ethanol and sodium acetate, dissolved in ddH_2_O DEPC (Thermo Fisher Scientific, Waltham, MA) and stored at −80°C. The absence of DNA contamination was verified with PCR via targeting the bacterial 16S rRNA gene (35 cycles; Haider *et al*. 2008). Ribosomal RNA was removed with the RiboZero Gold Magnetic kit as recommended by the manufacturer (Illumina, San Diego, CA). Quality control of purified RNA was performed using the Experion Automated Electrophoresis Station (Bio-Rad Laboratories, Hercules, CA). RNA integrity numbers (RIN) for all samples were higher than 8. All RNA isolations were performed in three biological independent triplicates, with RNA being isolated simultaneously from three infection experiments. RNA fragmentation was performed at 70°C for 5 min (RNA fragmentation reagents, Thermo Fisher Scientific). The NEBNext Ultra Directional RNA Library Prep Kit (Illumina) and NEBNext Multiplex Oligos (New England Biolabs, Ipswich, MA) were used for first-strand cDNA synthesis for a strand-specific cDNA library preparation. Libraries were sequenced on an Illumina HiSeq 2000 machine, with each library on an individual lane. Sequencing (50 bp read length, single-end configuration) was done at the Vienna Biocenter Core Facilities VBCF NGS Unit (www.vbcf.ac.at).

### Read processing and gene expression analysis

Reads were removed using mothur (Schloss *et al*. 2009) when the phred score decreased below 35 (window size = 10), when sequences were shorter than 30 bases or when reads contained more than eight homopolymers or an ambiguous base. All exact duplicate reads were removed to improve the strength of biological signals from high-throughput gene expression analyses (Dozmorov *et al*. 2015). To map bacterial reads to the *A. asiaticus* 5a2 reference genome (NC_010830.1; Schmitz-Esser *et al*. 2010), the BWA mapping tool (Li and Durbin 2009) was used with default settings. Reads per predicted gene were counted via ReadXplorer (Hilker *et al*. 2014) and analysed with the Bioconductor DeSeqE package using the R software (R_Development_Core_Team 2008). Differentially expressed genes were calculated using the negative binomial (NB) distribution model *K_ij_*∼NB(*μ_ij_*, *σ^2^_ij_*) with read counts *K_ij_*, mean *μ* and variance *σ^2^* (Anders and Huber 2010). After estimating size factors, we tested for differential expression by comparing two consecutive time points (12 h p.i. versus 72 h p.i., 72 h p.i. versus 144 h p.i., 144 h p.i. versus extracellular stage (EC) and EC versus 12 h p.i.; Anders and Huber 2010). Transcription values were listed as read counts per gene, with genes being considered differentially expressed if *P* < 0.05 and a FDR < 10% (Benjamini and Hochberg 1995).

To determine temporal gene expression patterns, hierarchical clustering of fold-changes (log_2_ fold-change) between two consecutive time points was done with all differentially expressed genes based on the hierarchical grouping Ward D method (Ward 1963) and visualized as heat maps using the R package dendextend (Galili 2015) and gplots (Warnes *et al*. 2009). The analysis of the *Acanthamoeba* host gene expression was not performed in this study.

### Data availability

The raw sequencing data were deposited at the NCBI Sequence Read Archive under Bioproject number PRJNA691613.

## Results and discussion

### General gene expression patterns

**Figure 1.**
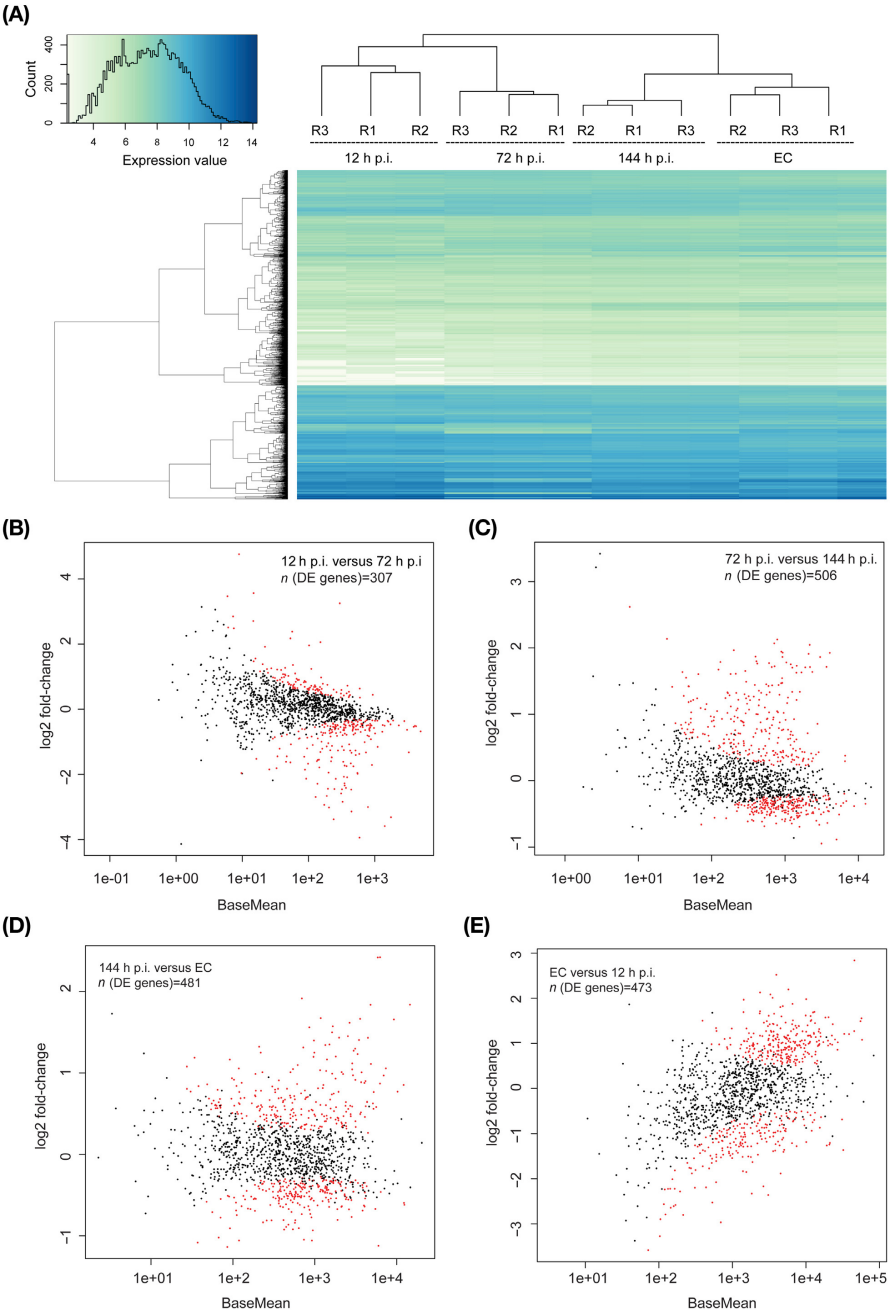
**(A)**. Euclidean clustering of replicates (R) 1–3 for all time points based on the gene expression levels of *A. asiaticus*.Expression values are given as transformed log_2_ values of normalized counts per gene (white = low expression and blue = high expression). EC = extracellular stage and h p.i. = hours post-infection. B–E. Plots of normalized mean counts (BaseMean) versus log_2_ fold-changes of gene expression along the life cycle of *A. asiaticus*. **(B)**. log_2_ fold-changes for the contrast 12 h p.i. versus 72 h p.i., **(C)**. log_2_ fold-changes for the contrast 72 h p.i. versus 144 h p.i., **(D)**. log_2_ fold-changes for the contrast 144 h p.i. versus EC and **(E)**. log_2_ fold-changes for the contrast EC versus 12 h p.i. Differentially expressed (DE) genes are colored red.

We performed RNA-Seq at an early (12 h), intermediate (72 h) and late (144 h) infection stage, respectively, as well as with symbionts released from amoeba host cells. Sequencing resulted in between 224 and 245 million reads per replicate (*n* = 12), from which 3.1–8.9% of the reads (mean length = 48 bp) remained after quality control and after removal of duplicate sequences (Table S1, Supporting Information). The percentage of trimmed unique reads mapped to the *A. asiaticus* genome (size: 1.9 Mbp) varied depending on the time point in the life cycle ranging from 0.8 to 5.8% at early time points to 13.2 to 37.9% at late time points. The difference between time points can be explained by distinct bacterial loads, reflecting the challenge to obtain high coverages of intracellular bacteria at early infection time points (Westermann, Gorski and Vogel 2012; Konig *et al*. 2017). Nevertheless, the percentage of the reference covered by at least one sequence read (Single Best Match Coverage) ranged from 64.5 to 99.6%. The mean theoretical redundancy of coverage was 6.3 × for 12 h p.i., 19.5 × for 72 h p.i., 75.5 × for 144 h p.i. and 64.9 × for the extracellular time point (Table S1, Supporting Information). These data reveal that the *A. asiaticus* gene transcription was adequately covered also during the early time points of infection. The number of expressed genes per time point was 99.9 to 100% of all predicted genes identified (Table S2, Supporting Information), and expression values within replicates were highly similar and reproducible (Fig. 1; Figure S2, Supporting Information).

To detect differentially expressed genes based on gene expression patterns during the *A. asiaticus* life cycle, gene expression patterns were determined for each time point. Differentially expressed genes were called between two consecutive time points (12 h p.i. versus 72 h p.i., 72 h p.i. versus 144 h p.i., 144 h p.i. versus EC and EC versus 12 h p.i.). It should be noted that differentially expressed genes could not be further validated using e.g. quantitative Reverse Transcriptase PCR (RT-PCR) because of the low amount of total RNA, but (Bock *et al*. 2017) evaluated the abundance of T6SS^iv^ arrays in *Amoebophilus* by electron cryotomography and it was found to be highest in extracellular and early intracellular infection stages, which is in accordance with the RNA-Seq data presented here. Recent publications show high correlations between RNA-Seq and quantitative RT-PCR (Consortium 2014; Everaert *et al*. 2017; Coenye 2021). Similar correlation results (regression *P* < 0.001; correlation coefficient *r* = 0.98) could be achieved in a recent study from our group (Mann *et al*. 2017), where we used the same RNAseq analysis tools for transcriptional analysis of *C. hertigii*, which provides additional evidence for the quantitative accuracy of the RNAseq data set.

**Figure 2.**
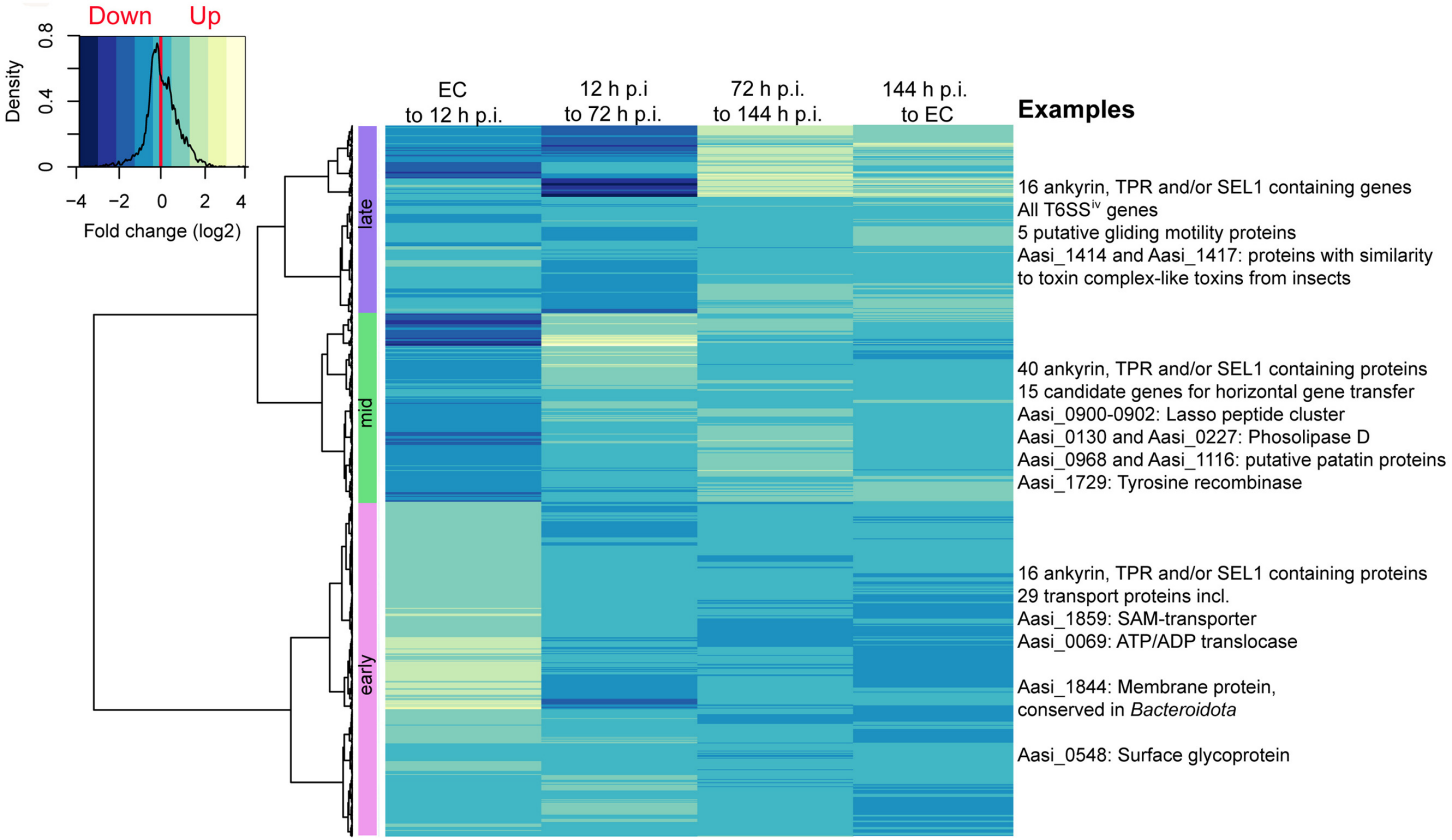
Temporal gene expression patterns during the *A. asiaticus* life cycle. Hierarchical clustering of fold-changes between two consecutive time points was done with all differentially expressed genes (*n* = 884) based on the Ward D method. Clustering identified three main temporal classes (early, mid and late genes) based on the dendrogram's branching hierarchy. Relevant examples are listed for each cluster. EC = extracellular stage and h p.i. = hours post-infection.

Genes without statistically significant shifts were considered constitutively expressed genes (*n* = 672). A total of four main clusters representing different constitutive expression levels were identified (Figure S3, Supporting Information). A total of 11 genes form the cluster of the highest expressed constitutive genes and comprised various housekeeping genes and five hypothetical genes, which included the most highly expressed constitutive gene Aasi_0654, a predicted lipoprotein that is only found in *A. asiaticus* (Table S3, Supporting Information). Other constitutively highly expressed genes included two proteins with ANK repeats (Aasi_0519 and Aasi_0225) and Aasi_1714, a putative surface protein showing similarity to adhesins from eukaryotic and bacterial pathogens (Penz, Horn and Schmitz-Esser 2010). In general, adhesin binding can be essential for initial attachment and is seen as a key mechanism for host cell invasion (Nizet, Varki and Aebi 2015). It has emerged during the last decade that some adhesins also directly affect host cell signaling to foster spreading and survival (Stones and Krachler 2015), and that for example, toxin secretion after invasion can be dependent on adhesins, as it still requires close physical contact to the host cell to enhance the efficiency of secretion because of low toxin concentrations (Kim *et al*. 2008; Ishijima *et al*. 2011). The constitutive expression during the life cycle of *A. asiaticus* could be a first hint that stable adhesin expression is crucial over the entire symbiont's life cycle and that adhesins have a dual function during and after colonization, contributing to a wide repertoire of effector activities (Stones and Krachler 2016). A total of 884 genes were differentially expressed and significantly up- or down-regulated at least once during the *A. asiaticus* life cycle (Fig. 1; Table S4, Supporting Information). The most highly expressed differentially expressed genes included many housekeeping genes involved in transcription and translation, such as RNA polymerase, elongation factors and ribosomal proteins. In addition, several genes predicted to be involved in host cell interaction were also among the most highly expressed differentially expressed genes (Table S5, Supporting Information). Overall, the log2 fold-changes across the entire *A. asiaticus* life cycle transcriptome ranged from −3.97 to +4.60 (Fig. 1). The log2 fold-changes of differentially expressed genes among the life cycle were clustered and three main clusters of gene expression could be defined (Fig. 2). The maximum of expression dynamics occurred early in development, peaking at 12 h p.i. (*n* = 417 genes). Mid-cycle expressed genes were defined with peaks at 72 and 144 h p.i. (*n* = 230) and late expressed genes with peaks at the extracellular stage (*n* = 230). A total of seven genes were excluded from clustering because at least one time point contained zero reads for all replicates.

### Early development of *A. asiaticus* within its *Acanthamoeba* host (EC–12 h p.i.)

**Figure 3.**
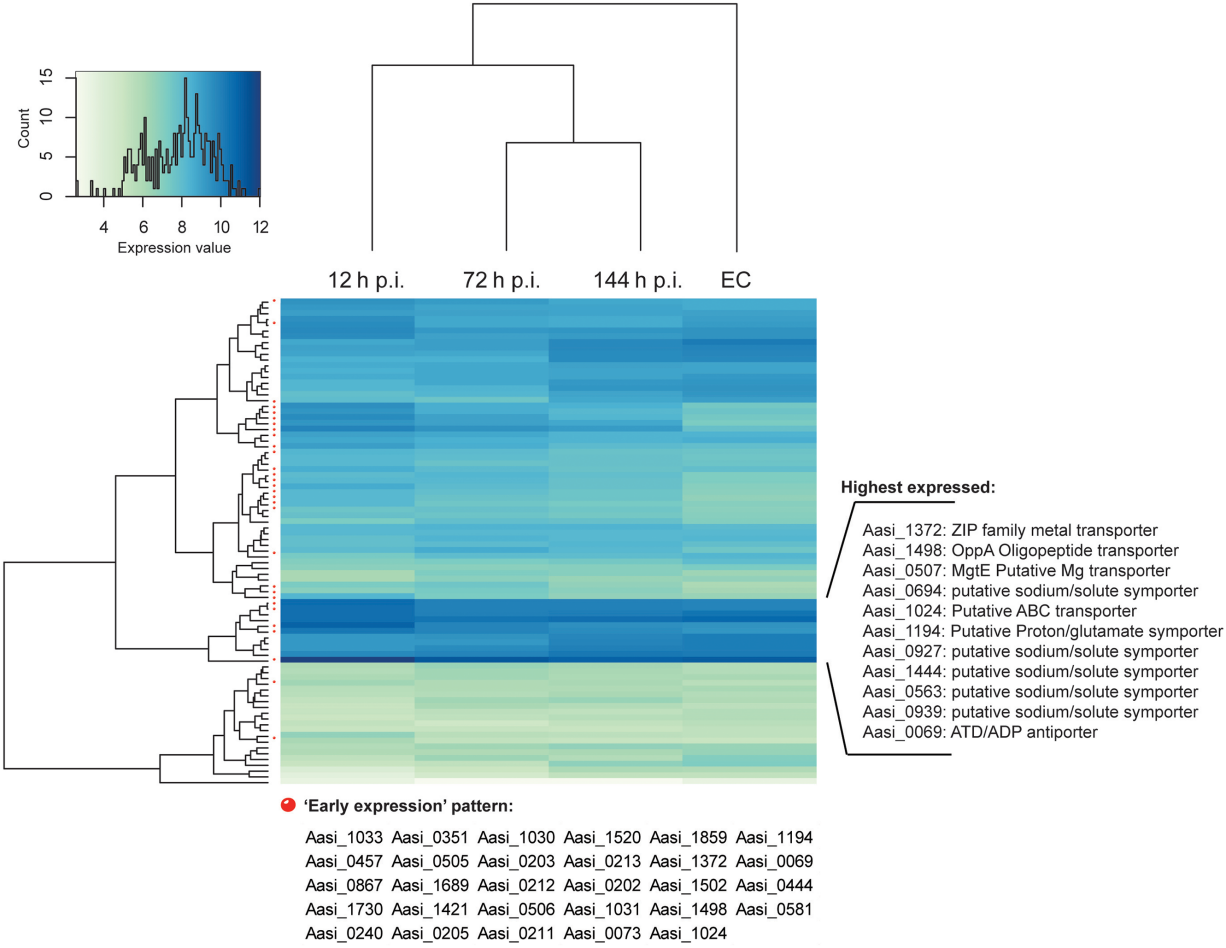
Hierarchical clustering of transporter gene expression (*n* = 84 genes). Genes that show an early expression pattern (Fig. 2) are marked with red dots. The locus_tags of the highest expressed transporters are listed.

The pronounced expression dynamics after the initial attachment and internalization phase (12 h p.i.) indicate the early establishment in the amoeba host cell and a high demand for gene expression adjustment during the transition from the EC stage to the intracellular environment. During this phase of the *A. asiaticus* life cycle, 274 genes were significantly upregulated. Among those, 29 transporters were upregulated at 12 h p.i. (Fig. 3), including Aasi_0069, a putative ATP/ADP translocase, which enables *Amoebophilus* to utilize the host's ATP pool. Similar antiporters are known from chlamydiae and rickettsiae (Schmitz-Esser *et al*. 2004; Audia and Winkler 2006; Major, Embley and Williams 2017). The high expression of the ATP/ADP transporter, thus likely supplements energy generation during this phase of internalization. The *Amoebophilus*-related insect symbiont *C. hertigii* also harbors a putative ATP/ADP transporter, which was found to be moderately expressed under natural conditions in its wasp host (Mann *et al*. 2017). The amoeba symbiont *Protochlamydia amoebophila* also shows high expression of a homologous ATP/ADP transporter during intracellular replication (Konig *et al*. 2017). Similarly, the functionally characterized *A. asiaticus* SAM carrier (Aasi_1859; Haferkamp *et al*. 2013) was highly upregulated during the early development. The expression of the SAM transporter, thus compensates for the inability of *A. asiaticus* to synthesize SAM, an essential cofactor for methylations. The *Cardinium* SAM transporter homolog was also expressed under natural conditions (Mann *et al*. 2017), suggesting that both the ATP/ADP and the SAM transporters are important for acquiring host-derived key metabolites in this lineage of symbionts. The upregulated transporters also included a putative oligopeptide/proton symporter (GltP, Aasi_1194), the OppACF oligopeptide/amino acid transporters and the DcuAB-like C_4_-dicarboxylate transporter (Aasi_0247). These transporters, thus likely provide the amino acids and oligopeptides that *A. asiaticus* needs for growth to compensate for the absence of complete *de novo* amino acid biosynthesis pathways in its genome, a feature found in many amoeba symbionts and also in the arthropod symbiont *Cardinium* (Konig *et al*. 2017; Mann *et al*. 2017; Wang and Wu 2017). The early upregulation of these transporters could also be related to an influx of energy (ATP), in order to facilitate early metabolic activity in preparation for cell division. The putative *A. asiaticus* biotin transporter *bioY* (Aasi_1496) was constitutively expressed.

**Figure 4.**
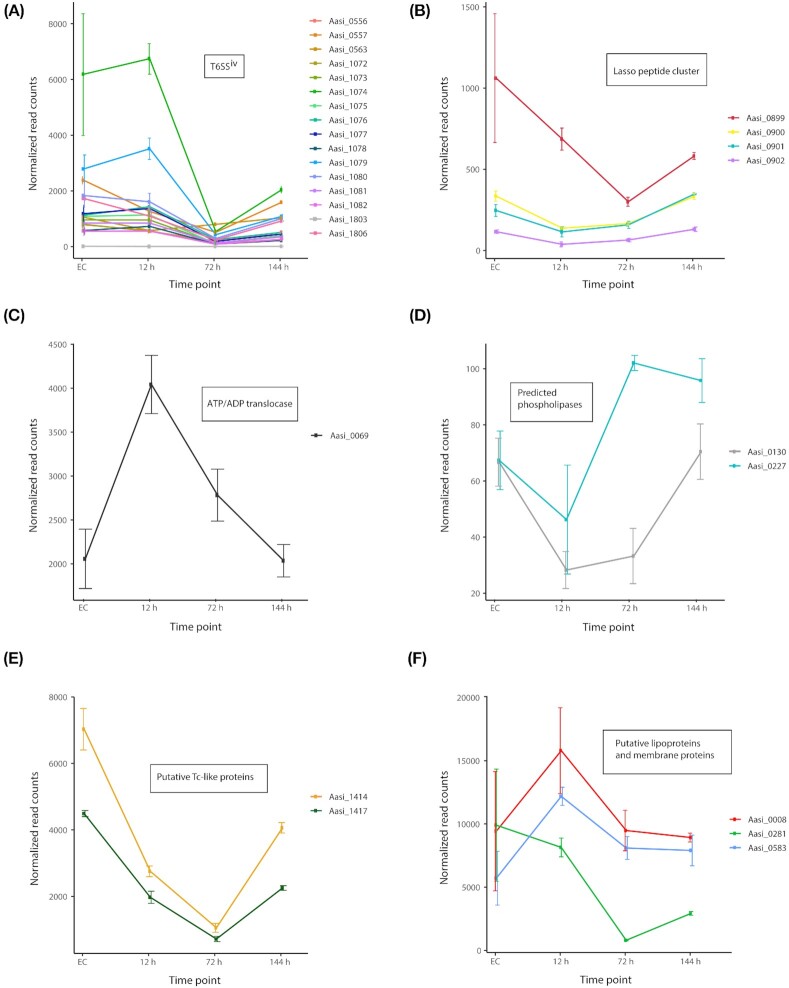
Expression levels of selected gene sets along the life cycle of *A. asiaticus*. Normalized read counts are shown, the error bars display the standard deviation. **(A)** Genes belonging to the type 6 secretion system (T6SS^iv^), **(B)** genes belonging to the putative Lasso peptide cluster, **(C)** shows gene expression dynamics of the ATP/ADP translocase, **(D)** shows predicted phospholipases, **(E)** shows Aasi_1414 and Aasi_1417, which have similarity to Tc-like toxins and **(F)** shows expression dynamics of the putative lipoproteins Aasi_0008 and Aasi_0281 and the predicted outer membrane protein Aasi_0583.

Other highly upregulated genes include Aasi_1844, a conserved membrane protein with unknown function found in many *Bacteroidota*, Aasi_0583, a predicted outer membrane protein and a putative cell surface protein (Aasi_0548) with a biased amino acid composition (increased serine and threonine content; Penz, Horn and Schmitz-Esser 2010). In addition, two predicted lipoproteins (Aasi_0008 and Aasi_0281) were significantly upregulated during this stage; Aasi_0008 was the highest overall expressed gene throughout the *A. asiaticus* life cycle, and Aasi_0281 was among the 20 most highly expressed genes except for the 144 h p.i. time point (Fig. 4; Tables S2 and S5, Supporting Information). Aasi_0583 was among the seven most highly expressed genes. The upregulation of these membrane and surface proteins suggests a reorganization of the *A. asiaticus* outer membrane and/or cell surface to aid in adaptation to the intracellular environment in the host cytoplasm. Several ELPs were upregulated during this time, including the two ANK repeat proteins Aasi_1610 and Aasi_0240, six TPR/SEL1 repeat proteins, one gene harboring an F-Box domain and Aasi_1805 and one of the two predicted ubiquitin-specific proteases found in the *A. asiaticus* genome. The significant upregulation of ELPs suggests that these proteins are important for manipulating host cellular pathways to establish the intracellular niche of *A. asiaticus*.

In total, 199 genes were significantly downregulated during the early development of *A. asiaticus*. Recently, a novel subtype of type 6 secretion systems (T6SS^iv^) consisting of 18 structural genes was functionally characterized in *A. asiaticus* (Bock *et al*. 2017); three of these T6SS^iv^ genes were significantly downregulated. Aasi_1414 and Aasi_1417, which show similarity to insecticidal toxins (Tc toxin complex) from *Photorhabdus* (Schmitz-Esser *et al*. 2010), were downregulated as well (see below for more details on these proteins). *Amoebophilusasiaticus* contains 19 putative sodium/solute symporters, and 10 of these transporter genes were downregulated at this life cycle stage. The function of these putative transporters is currently unknown; functionally characterized sodium/solute symporters are known to be involved in the uptake of various nutrients (Jung 2002). This lets us conclude that *A. asiaticus* clearly prioritize transcription of genes important for the transition from the EC stage to the intracellular environment at this time point, and that the acquisition of certain nutrients is more important at later time points.

### Replicative and host cell lysing-stage of *A. asiaticus*

#### 12–72 h p.i

This phase of the *A. asiaticus* life cycle is characterized by phagosome escape and differentiation from coccoid cells to rods, and increasing replication inside the *Acanthamoeba* host cell cytoplasm (Bock *et al*. 2017). A total number of 307 genes were differentially expressed between the 12 and 72 h p.i. time points. This included 102 and 205 genes being up- or down-regulated, respectively. Among the upregulated genes were nine ANK repeat proteins, two F-box domain proteins and 16 TPR-SEL1 repeat proteins. Aasi_0227, a predicted phospholipase D was also significantly upregulated (Fig. 4); Aasi_0227 is highly similar to the *Rickettsia* phospholipase D, which has been shown to be important for vacuole escape (Whitworth *et al*. 2005). This indicates that this phospholipase is—possibly in concert with the T6SS^iv^—involved in the escape from the vacuole, as *A. asiaticus* is located directly in the host cell cytosol and not surrounded by host-derived membranes (Horn *et al*. 2001; Schmitz-Esser *et al*. 2008, 2010).

Among the downregulated genes from this life cycle stage were 15 of the T6SS^iv^ genes, four ANK repeat proteins and 10 TPR/SEL1 repeat proteins. The putative cell surface protein Aasi_0548 was downregulated similar to the ATP/ADP translocase Aasi_0069, as was Aasi_1805, the putative ubiquitin-specific protease. Interestingly, most of these genes were upregulated at 12 h p.i., thus providing additional evidence for their role during early infection of amoebae.

#### 72–144 h p.i

During the later stage of intracellular replication of *A. asiaticus*, the amoebae become completely filled with *A. asiaticus* around 120–144 h p.i. and *A. asiaticus* ultimately lyses its host cells (Bock *et al*. 2017). A total number of 506 genes were differentially expressed between these two time points, including 267 upregulated and 239 downregulated genes. Among the upregulated genes were 16 ANK repeat proteins, six F-/U-Box domain proteins, four LRR repeat proteins and 26 TPR-SEL1 repeat proteins. Additionally, Aasi_0130, another predicted phospholipase D, showing high similarity to the *Rickettsia* phospholipase D and to Aasi_0227, was upregulated (Fig. 4). The upregulation of a different putative phospholipase D suggests that the two phospholipase D genes in the *A. asiaticus* genome fulfill distinct functions during its life cycle. Aasi_1414 and Aasi_1417, which show similarity to the Tc toxin complex family, were significantly upregulated. Aasi_1414 and Aasi_1417 showed the highest expression levels at the 144 h p.i and EC time points where they were among the top 25 and top 10 of the most highly expressed genes, respectively (Fig. 4; Tables S2 and S5, Supporting Information). Tc toxins are virulence factors of many bacteria, including insect and human pathogens. These proteins perforate the host membrane and translocate effectors into the host cell cytosol (Roderer and Raunser 2019). As Aasi_1414 and Aasi_1417 show homology to the three characteristic Tc toxin subunits TcA, TcB and TcC, it is tempting to speculate that the *A. asiaticus* Tc toxin homologs function in a similar way to deliver effector proteins into the *Acanthamoeba* host cell cytosol or possibly also to the nucleus. Interestingly, the highly conserved region in TcC is also conserved in Aasi_1414, including the catalytic aspartate residues of the TcC autoprotease domain (Meusch *et al*. 2014; Roderer *et al*. 2019; data not shown). The *A. asiaticus* Tc-like proteins have no homologs in other amoeba symbionts or in the insect symbiont *C. hertigii*. Thus, they represent a specific adaptation of *A. asiaticus* to its particular lifestyle and amoeba host. The specific function of Aasi_1414 and Aasi_1417 will need to be investigated in future studies.

In contrast to the early time points, 15 of the T6SS^iv^ genes were significantly upregulated at this time point, suggesting the formation of the T6SS^iv^ during this life cycle stage. This is consistent with a significant upregulation of the T6SS^iv^ sheath protein (Aasi_1074) gene demonstrated previously (Bock *et al*. 2017). We previously identified a gene cluster (Aasi_0899–Aasi_0902) encoding a putative lasso peptide in the *A. asiaticus* genome (Schmitz-Esser *et al*. 2010), which was upregulated here. Lasso peptides are ribosomally synthesized peptides that can have various biological functions, including antimicrobial and anti-host cell activities (Hegemann *et al*. 2015). Interestingly, homologs with high similarity (50–61% amino acid identity) to the *A. asiaticus* lasso peptide gene cluster are also present in the genomes of the alphaproteobacterial amoeba symbionts *Paracaedibacter symbiosus* and *Odyssella thessalonicensis*, both of which are also found directly in the host cell cytosol (Horn *et al*. 1999; Birtles *et al*. 2000). Thus, the lasso peptide gene cluster of amoeba symbionts may have an important yet unknown function in host cell interaction. A total of 10 of the 19 putative sodium/solute symporter genes were upregulated at this life cycle stage. A total of five of the putative sodium/solute symporters were among a cluster of the 11 highest expressed transporters (Fig. 3). Although the substrate specificities of these putative sodium/solute symporters are currently unknown, the presence of a high number of these putative transporters and their expression patterns suggest that these transporters fulfill an important function in acquisition of nutrients such as proline, galactose, glucose, pantothenate or nucleosides by *A. asiaticus* at the later time points. Also, *C. hertigii* harbors 12 putative sodium/solute symporters, which were also found to be expressed under natural conditions (Mann *et al*. 2017).

Among the significantly downregulated genes were several ELPs, including three ANK repeat proteins and two F-Box and TPR-SEL1 repeat proteins. Similar to the 12–72 h p.i. life cycle stage, the putative cell surface protein Aasi_0548 was also downregulated, as was the ATP/ADP translocase Aasi_0069.

### Late and extracellular stage of *A. asiaticus* (144 h p.i.–EC)

This stage of the *A. asiaticus* life cycle is characterized by host cell lysis and release of *A. asiaticus* and the highest presence of T6SS^iv^ system structures (Bock *et al*. 2017). A total number of 481 genes were differentially expressed between these two time points with 250 upregulated and 231 downregulated genes. Among the upregulated genes were seven ANK repeat proteins, six F-/U-Box domain proteins, two leucine-rich repeat (LRR) proteins and 10 TPR-SEL1 repeat proteins. Similar to the 72–144 h p.i. life cycle stage, 15 of the T6SS^iv^ system genes were also upregulated. In fact, the T6SS^iv^ genes showed the highest expression values during this phase of the *A. asiaticus* life cycle (Fig. 4; Tables S2 and S5, Supporting Information). Recently, a significant upregulation of the T6SS^iv^ sheath protein gene (Aasi_1074) was demonstrated using quantitative RT-PCR for *A. asiaticus* during its EC life cycle phase (Bock *et al*. 2017). This is in accordance with our RNA-seq dataset, where Aasi_1074 was significantly upregulated during the EC stage (3-fold change compared to 144 h p.i.).

*Cardinium hertigii*, the sister lineage of *A. asiaticus*, also harbors a T6SS^iv^; recently, we showed that the T6SSiv is also highly expressed in *Cardinium*(Mann *et al*. 2017). However, no temporal transcriptome data are currently available for *Cardinium*. Although substrates of the T6SS^iv^ are currently unknown, the expression pattern of the T6SS^iv^ suggests that it is crucial for host cell interaction either at the late and extracellular stages or also for phagosome exit of the *A. asiaticus* life cycle. Related secretion systems such as the metamorphosis-associated contractile (MAC) arrays of the symbiont *Pseudoalteromonas luteoviolaceais* or the *Serratia* antifeeding prophage (AFP) use a similar contractile sheath-rigid tube mechanism to puncture the eukaryotic cell envelope, and are responsible for the induction of metamorphosis of the tubeworm *Hydroides elegans* (Shikuma *et al*. 2014; Ericson *et al*. 2019) or deliver toxins into insect larvae (Hurst, Glare and Jackson 2004). Interestingly, T6SS^iv^ homologs have also been found in *Bacteroidota* members in the human microbiota (Rojas *et al*. 2020), providing further evidence for the widespread presence of these contractile secretion systems. Future experiments will be needed to identify the substrates of the *A. asiaticus* T6SS^iv^. It should be noted that *A. asiaticus* is the only amoeba symbiont currently known to harbor a T6SS^iv^; in contrast, other symbionts of free-living amoebae, such as *Chlamydia*-related amoeba symbionts, encode a type 3 secretion system to deliver effectors into their eukaryotic host cells (Mueller, Plano and Fields 2014; Collingro, Kostlbacher and Horn 2020). The seven ANK repeat proteins, six of the TPR/SEL1 repeat proteins and two of the LRR proteins which were upregulated here were also upregulated from 72 to 144 h p.i.. In total, two of the putative sodium/solute symporters which were upregulated from 72 to 144 h p.i. were also upregulated here. Similar to the previous phase of the life cycle, the Tc toxin homologs Aasi_1414 and Aasi_1417, as well as Aasi_0899, the putative toxin from the putative lasso peptide cluster, were upregulated during this phase. These consistent upregulation patterns of the aforementioned genes suggest that these genes are either important throughout the late and EC life cycle stages of *A. asiaticus*; alternatively, these genes could also be required during the initial infection stages in a way that *A. asiaticus* is ‘armed’ for infection.

The phospholipase D Aasi_0227 was significantly downregulated during this life cycle period, providing further evidence for the life-cycle-specific expression of the two different *A. asiaticus* phospholipases. Among ELPs, six ANK repeat proteins, three F/U-box domain proteins and seven TPR/SEL1 repeat proteins were downregulated. The downregulation of these genes suggests that they might be important to maintain the intracellular niche, e.g. by interacting with host pathways during *A. asiaticus* intracellular replication.

### Expression of transposase genes

One remarkable feature of the *A. asiaticus* 5a2 genome is the presence of very high numbers of transposase genes. The *A. asiaticus* 5a2 genome encodes 354 transposase genes representing 23% of all coding sequences (Schmitz-Esser *et al*. 2010, 2011). These transposases were previously classified into 15 IS elements found in varying copy numbers in the *A. asiaticus* genome (Schmitz-Esser *et al*. 2011). In total, 352 of these transposase genes present in *A. asiaticus* were transcribed at least at one time point of the *A. asiaticus* life cycle, with 74 transposases identified as differentially expressed genes. Overall, the expression level of the transposase genes was low to medium. The expression of many transposases has been observed in other recent studies in endosymbionts. They have been suggested to play a role in transposable element expansion and to be important for the evolution of these bacteria (Kleiner *et al*. 2013; Sanders *et al*. 2013). We also observed high levels of transposase gene expression in *C. hertigii*, where we found more than half of the transposases to be expressed (Mann *et al*. 2017). However, we showed previously that despite transcriptional activity, the IS elements in *A. asiaticus* 5a2 were transpositionally inactive (Schmitz-Esser *et al*. 2011). Thus, the relevance of transposase gene expression in *A. asiaticus* remains to be elucidated. It is known that the insertion of IS elements upstream of a gene can activate gene expression of the downstream gene (Siguier, Gourbeyre and Chandler 2014). Therefore, the observed transpositional inactivity of the *A. asiaticus* IS elements might partially be explained by IS elements influencing neighboring gene expression.

### The potential role of ELPs in host cell interaction

The importance of ELPs for host cell interaction has been demonstrated for a number of bacteria, including bacterial symbionts and human pathogens (Gomez-Valero *et al*. 2011; Frank 2019; Mondino, Schmidt and Buchrieser 2020). The occurrence of genes harboring eukaryotic domains in bacterial genomes has been termed molecular mimicry (Frank 2019; Mondino, Schmidt and Buchrieser 2020). In this way, bacterial pathogens and intracellular symbionts utilize ELPs to imitate certain host proteins to manipulate host pathways. Genes harboring eukaryotic domains include LRRs, which are involved in protein–protein interactions like cell adhesion or invasion to host cells (Bierne *et al*. 2007; Bella *et al*. 2008). Another group of ELPs that has been shown to be important for host cell interaction are TPR/SEL1 repeat proteins (Cerveny *et al*. 2013). TPR/SEL1 repeat proteins from bacterial sponge symbionts have been shown to mediate amoeba interactions when expressed in *Escherichia coli* (Reynolds and Thomas 2016). Expression of TPR/SEL1 repeat proteins has also been observed in sponge symbionts using metatranscriptome sequencing (Diez-Vives *et al*. 2017). Similarly, proteins containing ANK repeats can mediate protein–protein interactions, thereby influencing eukaryotic transcription, cell signaling, bacterial invasion and many more and they can be found in many bacterial pathogens and intracellular symbionts, including *Legionella* (Li, Mahajan and Tsai 2006; Voth 2011; Mondino, Schmidt and Buchrieser 2020). ANK repeat proteins from different sponge symbionts have been shown to modulate the host response against bacteria (Jahn *et al*. 2019) and are involved in the internalization of bacteria in amoebae (Nguyen, Liu and Thomas 2014; Reynolds and Thomas 2016). Ubiquitination and deubiquitination of proteins regulate many eukaryotic cellular processes. A number of bacterial proteins have been shown to interact with the ubiquitination system of eukaryotic cells (Zhou and Zhu 2015; Ashida and Sasakawa 2017; Kubori, Kitao and Nagai 2019). This includes proteins harboring F-box and U-box domains, ubiquitin ligases and putative ubiquitin-specific proteases. We have previously shown that *A. asiaticus* encodes an unusually high number of genes (*n* = 129) harboring eukaryotic domains, including LRR proteins, TPR/SEL1 repeat proteins, ANK repeat proteins and 26 proteins predicted to interact with the host ubiquitin system (Schmitz-Esser *et al*. 2010). Here, we reveal the expression of all of these genes during at least one time point of the *A. asiaticus* life cycle. This generally high expression level and the differential expression of many of these genes with eukaryotic domains suggest that these genes are important for *A. asiaticus* interaction with its amoebal host. Additionally, the specific up- and down-regulation of genes harboring eukaryotic domains throughout the different stages of the *A. asiaticus* life cycle (Figures S4, S5 and S6, Supporting Information) suggest that these genes harboring eukaryotic domains have different and highly specific functions throughout the *A. asiaticus* life cycle, despite sharing the repeats. Also, in the insect symbiont *C. hertigii* living in its natural host environment, we observed high expression levels of genes harboring eukaryotic domains, including ANK, TPR-SEL1-repeats and F- and U-Box domains (Mann *et al*. 2017). The chlamydial amoeba symbiont *P. amoebophila* also shows high expression levels of genes harboring eukaryotic domains, particularly at early time points in the life cycle, and among them are a great number of genes harboring TPR-SEL1-repeats and genes that are potentially involved in the modification of host ubiquitination (Konig *et al*. 2017). Expansion of ubiquitination-related gene families has also been observed in protozoa-associated members of the *Parachlamydiaceae* family within the *Chlamydiae* phylum (Domman *et al*. 2014). In line with this, also in bacterial sponge symbionts, high expression levels and differential expression of ELPs were observed (Diez-Vives *et al*. 2017). Alternatively, the high number of different ELPs in *A. asiaticus* may also represent an adaptation to different hosts, such as various protists.

Although several genes harboring eukaryotic domains have been functionally characterized, e.g. in *L. pneumophila*, which also has an intra-amoebal life cycle stage (Mondino, Schmidt and Buchrieser 2020), the function of the many *A. asiaticus* genes with eukaryotic domains remains uncharacterized until now. Future experimental studies will be needed to verify the function of these proteins in *A. asiaticus* host cell interaction.

Our transcriptome sequencing data reveals comprehensive insights into the lifestyle of these elusive bacteria and shows massive expression of ELPs and a novel, widespread variant of T6SS and reveals possibly important candidate genes for host cell interaction. The results from this study shed new light on the host cell interaction mechanism in the lineage of *Amoebophilaceae* harboring arthropod and amoeba symbionts. They will also be useful to elucidate general and specific host cell interaction mechanisms of amoeba-associated bacteria belonging to other bacterial phyla.

## Supplementary Material

fiac001_Supplemental_Files

## References

[bib1] Alteio LV , Schulz F, Seshadri R et al. Complementary metagenomic approaches improve reconstruction of microbial diversity in a forest soil. mSystems. 2020;5:e00768–19. 32156798 10.1128/mSystems.00768-19 PMC7065516

[bib2] Anders S , Huber W. Differential expression analysis for sequence count data. Genome Biol. 2010;11:R106. 20979621 10.1186/gb-2010-11-10-r106 PMC3218662

[bib3] Apprill A , Weber LG, Santoro AE. Distinguishing between microbial habitats unravels ecological complexity in coral microbiomes. mSystems. 2016;1:e00143–16. 27822559 10.1128/mSystems.00143-16 PMC5080407

[bib4] Ashida H , Sasakawa C. Bacterial E3 ligase effectors exploit host ubiquitin systems. Curr Opin Microbiol. 2017;35:16–22. 27907841 10.1016/j.mib.2016.11.001

[bib5] Audia JP , Winkler HH. Study of the five *Rickettsia**prowazekii* proteins annotated as ATP/ADP translocases (Tlc): only Tlc1 transports ATP/ADP, while Tlc4 and Tlc5 transport other ribonucleotides. J Bacteriol. 2006;188:6261–8. 16923893 10.1128/JB.00371-06 PMC1595366

[bib6] Bandyopadhyay P , Sumer EU, Jayakumar D et al. Implication of proteins containing tetratricopeptide repeats in conditional virulence phenotypes of *Legionella**pneumophila*. J Bacteriol. 2012;194:3579–88. 22563053 10.1128/JB.00399-12 PMC3393475

[bib7] Bella J , Hindle KL, McEwan PA et al. The leucine-rich repeat structure. Cell Mol Life Sci. 2008;65:2307–33. 18408889 10.1007/s00018-008-8019-0 PMC11131621

[bib8] Benjamini Y , Hochberg Y. Controlling the false discovery rate - a practical and powerful approach to multiple testing. J Roy Stat Soc B Met. 1995;57:289–300.

[bib9] Bierne H , Sabet C, Personnic N et al. Internalins: a complex family of leucine-rich repeat-containing proteins in *Listeria**monocytogenes*. Microbes Infect. 2007;9:1156–66. 17764999 10.1016/j.micinf.2007.05.003

[bib10] Birtles RJ , Rowbotham TJ, Michel R et al.‘Candidatus *Odyssella thessalonicensis*’ gen. nov., sp. nov., an obligate intracellular parasite of Acanthamoeba species. Int J Syst Evol Microbiol. 2000;50:63–72. 10826788 10.1099/00207713-50-1-63

[bib11] Bock D , Medeiros JM, Tsao HF et al. In situ architecture, function, and evolution of a contractile injection system. Science. 2017;357:713–7. 28818949 10.1126/science.aan7904 PMC6485382

[bib12] Brock DA , Noh S, Hubert ANM et al. Endosymbiotic adaptations in three new bacterial species associated with *Dictyostelium**discoideum*: *Paraburkholderia**agricolaris* sp. nov., *Paraburkholderia**hayleyella* sp. nov., and *Paraburkholderia**bonniea* sp. nov. PeerJ. 2020;8:e9151. 32509456 10.7717/peerj.9151 PMC7247526

[bib13] Cerveny L , Straskova A, Dankova V et al. Tetratricopeptide repeat motifs in the world of bacterial pathogens: role in virulence mechanisms. Infect Immun. 2013;81:629–35. 23264049 10.1128/IAI.01035-12 PMC3584863

[bib14] Choi SH , Cho MK, Ahn SC et al. Endosymbionts of *Acanthamoeba* isolated from domestic tap water in Korea. Korean J Parasitol. 2009;47:337–44. 19967080 10.3347/kjp.2009.47.4.337 PMC2788711

[bib15] Coenye T . Do results obtained with RNA-sequencing require independent verification?. Biofilm. 2021;3:100043. 33665610 10.1016/j.bioflm.2021.100043 PMC7823214

[bib16] Collingro A , Kostlbacher S, Horn M. Chlamydiae in the environment. Trends Microbiol. 2020;28:877–88. 32591108 10.1016/j.tim.2020.05.020

[bib17] Consortium SM-I . A comprehensive assessment of RNA-seq accuracy, reproducibility and information content by the sequencing quality control consortium. Nat Biotechnol. 2014;32:903–14. 25150838 10.1038/nbt.2957 PMC4321899

[bib18] Diez-Vives C , Moitinho-Silva L, Nielsen S et al. Expression of eukaryotic-like protein in the microbiome of sponges. Mol Ecol. 2017;26:1432–51. 28036141 10.1111/mec.14003

[bib19] Domman D , Collingro A, Lagkouvardos I et al. Massive expansion of Ubiquitination-related gene families within the Chlamydiae. Mol Biol Evol. 2014;31:2890–904. 25069652 10.1093/molbev/msu227 PMC4209131

[bib20] Dozmorov MG , Adrianto I, Giles CB et al. Detrimental effects of duplicate reads and low complexity regions on RNA- and ChIP-seq data. BMC Bioinf. 2015;16:S10. 10.1186/1471-2105-16-S13-S10 PMC4597324 26423047

[bib21] Ericson CF , Eisenstein F, Medeiros JM et al. A contractile injection system stimulates tubeworm metamorphosis by translocating a proteinaceous effector. eLife. 2019;8:e46845. 31526475 10.7554/eLife.46845 PMC6748791

[bib22] Everaert C , Luypaert M, Maag JLV et al. Benchmarking of RNA-sequencing analysis workflows using whole-transcriptome RT-qPCR expression data. Sci Rep. 2017;7:1559. 28484260 10.1038/s41598-017-01617-3 PMC5431503

[bib23] Frank AC . Molecular host mimicry and manipulation in bacterial symbionts. FEMS Microbiol Lett. 2019;366. DOI: 10.1093/femsle/fnz038. 30877310

[bib24] Galili T . dendextend: an R package for visualizing, adjusting and comparing trees of hierarchical clustering. Bioinformatics. 2015;31:3718–20. 26209431 10.1093/bioinformatics/btv428 PMC4817050

[bib25] Gomez-Valero L , Rusniok C, Cazalet C et al. Comparative and functional genomics of *Legionella* identified eukaryotic like proteins as key players in host–pathogen interactions. Front Microbiol. 2011;2:208. 22059087 10.3389/fmicb.2011.00208 PMC3203374

[bib26] Greub G , Raoult D. Microorganisms resistant to free-living amoebae. Clin Microbiol Rev. 2004;17:413–33. 15084508 10.1128/CMR.17.2.413-433.2004 PMC387402

[bib27] Haferkamp I , Penz T, Geier M et al. The endosymbiont *Amoebophilus**asiaticus* encodes an S-adenosylmethionine carrier that compensates for its missing methylation cycle. J Bacteriol. 2013;195:3183–92. 23667233 10.1128/JB.00195-13 PMC3697640

[bib28] Haider S , Collingro A, Walochnik J et al. Chlamydia-like bacteria in respiratory samples of community-acquired pneumonia patients. FEMS Microbiol Lett. 2008;281:198–202. 18312573 10.1111/j.1574-6968.2008.01099.x

[bib29] Haselkorn TS , Jimenez D, Bashir U et al. Novel Chlamydiae and Amoebophilus endosymbionts are prevalent in wild isolates of the model social amoeba *Dictyostelium discoideum*. Environ Microbiol Rep. 2021;13:708–19. 34159734 10.1111/1758-2229.12985 PMC8518690

[bib30] Hegemann JD , Zimmermann M, Xie X et al. Lasso peptides: an intriguing class of bacterial natural products. Acc Chem Res. 2015;48:1909–19. 26079760 10.1021/acs.accounts.5b00156

[bib31] Hilker R , Stadermann KB, Doppmeier D et al. ReadXplorer–visualization and analysis of mapped sequences. Bioinformatics. 2014;30:2247–54. 24790157 10.1093/bioinformatics/btu205 PMC4217279

[bib33] Horn M , Fritsche TR, Gautom RK et al. Novel bacterial endosymbionts of *Acanthamoeba**spp*. related to the *Paramecium**caudatum* symbiont *Caedibacter**caryophilus*. Environ Microbiol. 1999;1:357–67. 11207753 10.1046/j.1462-2920.1999.00045.x

[bib34] Horn M , Harzenetter MD, Linner T et al. Members of the Cytophaga-Flavobacterium-Bacteroides phylum as intracellular bacteria of acanthamoebae: proposal of ‘Candidatus Amoebophilus asiaticus'. Environ Microbiol. 2001;3:440–9. 11553234 10.1046/j.1462-2920.2001.00210.x

[bib32] Horn M , Wagner M. Bacterial endosymbionts of free-living amoebae. J Eukaryot Microbiol. 2004;51:509–14. 15537084 10.1111/j.1550-7408.2004.tb00278.x

[bib35] Huggett MJ , Apprill A. Coral microbiome database: integration of sequences reveals high diversity and relatedness of coral-associated microbes. Environ Microbiol Rep. 2019;11:372–85. 30094953 10.1111/1758-2229.12686 PMC7379671

[bib36] Hurst MR , Glare TR, Jackson TA. Cloning *Serratia**entomophila* antifeeding genes–a putative defective prophage active against the grass grub *Costelytra**zealandica*. J Bacteriol. 2004;186:5116–28. 15262948 10.1128/JB.186.15.5116-5128.2004 PMC451664

[bib37] Ishijima N , Suzuki M, Ashida H et al. BabA-mediated adherence is a potentiator of the *Helicobacter**pylori* type IV secretion system activity. J Biol Chem. 2011;286:25256–64. 21596743 10.1074/jbc.M111.233601 PMC3137096

[bib38] Jahn MT , Arkhipova K, Markert SM et al. A phage protein aids bacterial symbionts in eukaryote immune evasion. Cell Host Microbe. 2019;26:542–50. 31561965 10.1016/j.chom.2019.08.019

[bib39] Jernigan KK , Bordenstein SR. Ankyrin domains across the tree of life. PeerJ. 2014;2:e264. 24688847 10.7717/peerj.264 PMC3932732

[bib40] Jung H . The sodium/substrate symporter family: structural and functional features. FEBS Lett. 2002;529:73–7. 12354616 10.1016/s0014-5793(02)03184-8

[bib41] Kim YR , Lee SE, Kook H et al. *Vibrio vulnificus* RTX toxin kills host cells only after contact of the bacteria with host cells. Cell Microbiol. 2008;10:848–62. 18005241 10.1111/j.1462-5822.2007.01088.x

[bib42] Kleiner M , Young JC, Shah M et al. Metaproteomics reveals abundant transposase expression in mutualistic endosymbionts. mBio. 2013;4:e00223–00213. 23781067 10.1128/mBio.00223-13 PMC3684830

[bib43] Konig L , Siegl A, Penz T et al. Biphasic metabolism and host interaction of a chlamydial symbiont. mSystems. 2017;2:e00202–16. 28593198 10.1128/mSystems.00202-16 PMC5451489

[bib44] Kubori T , Kitao T, Nagai H. Emerging insights into bacterial deubiquitinases. Curr Opin Microbiol. 2019;47:14–9. 30391778 10.1016/j.mib.2018.10.001

[bib45] Lagkouvardos I , Shen J, Horn M. Improved axenization method reveals complexity of symbiotic associations between bacteria and acanthamoebae. Environ Microbiol Rep. 2014;6:383–8. 24992537 10.1111/1758-2229.12162

[bib46] Li H , Durbin R. Fast and accurate short read alignment with Burrows–Wheeler transform. Bioinformatics. 2009;25:1754–60. 19451168 10.1093/bioinformatics/btp324 PMC2705234

[bib47] Li J , Mahajan A, Tsai MD. Ankyrin repeat: a unique motif mediating protein–protein interactions. Biochemistry. 2006;45:15168–78. 17176038 10.1021/bi062188q

[bib48] Lomma M , Dervins-Ravault D, Rolando M et al. The *Legionella**pneumophila* F-box protein lpp2082 (AnkB) modulates ubiquitination of the host protein parvin b and promotes intracellular replication. Cell Microbiol. 2010;12:1272–91. 20345489 10.1111/j.1462-5822.2010.01467.x

[bib49] Major P , Embley TM, Williams TA. Phylogenetic diversity of NTT nucleotide transport proteins in free-living and parasitic bacteria and eukaryotes. Genome Biol Evolut. 2017;9:480–7. 10.1093/gbe/evx015 PMC5381601 28164241

[bib50] Mann E , Stouthamer CM, Kelly SE et al. Transcriptome sequencing reveals novel candidate genes for *Cardinium**hertigii*-caused cytoplasmic incompatibility and host-cell interaction. mSystems. 2017;2:e00141–17. 10.1128/mSystems.00141-17 PMC5698495 29181449

[bib51] Meusch D , Gatsogiannis C, Efremov RG et al. Mechanism of Tc toxin action revealed in molecular detail. Nature. 2014;508:61–5. 24572368 10.1038/nature13015

[bib52] Mittl PR , Schneider-Brachert W. Sel1-like repeat proteins in signal transduction. Cell Signal. 2007;19:20–31. 16870393 10.1016/j.cellsig.2006.05.034

[bib53] Molmeret M , Horn M, Wagner M et al. Amoebae as training grounds for intracellular bacterial pathogens. Appl Environ Microbiol. 2005;71:20–8. 15640165 10.1128/AEM.71.1.20-28.2005 PMC544274

[bib54] Mondino S , Schmidt S, Buchrieser C. Molecular mimicry: a paradigm of host–microbe coevolution illustrated by *Legionell**a*. mBio. 2020;11:e01201–20. 33024033 10.1128/mBio.01201-20 PMC7542358

[bib55] Mueller KE , Plano GV, Fields KA. New frontiers in type III secretion biology: the chlamydia perspective. Infect Immun. 2014;82:2–9. 24126521 10.1128/IAI.00917-13 PMC3911841

[bib56] Nguyen MT , Liu M, Thomas T. Ankyrin-repeat proteins from sponge symbionts modulate amoebal phagocytosis. Mol Ecol. 2014;23:1635–45. 23980812 10.1111/mec.12384

[bib57] Nizet V , Varki A, Aebi M. Microbial lectins: hemagglutinins, adhesins, and toxins. In: Varki A, Cummings RD (eds). Essentials of Glycobiology, New York, NY: Cold Spring Harbor, 2015, 481–91. 28876816

[bib59] Penz T , Horn M, Schmitz-Esser S. The genome of the amoeba symbiont “Candidatus Amoebophilus asiaticus” encodes an afp-like prophage possibly used for protein secretion. Virulence. 2010;1:541–5. 21178499 10.4161/viru.1.6.13800

[bib58] Penz T . Genomic insights into molecular interactions of two bacteroidetes symbionts with their eukaryotic hosts. Ph.D. Thesis. University of Vienna, 2014.

[bib60] Price CT , Al-Quadan T, Santic M et al. Host proteasomal degradation generates amino acids essential for intracellular bacterial growth. Science. 2011;334:1553–7. 22096100 10.1126/science.1212868

[bib61] R_Development_Core_Team . R: A Language and Environment for Statistical Computing. R Foundation for Statistical Computing, Vienna, Austria, 2008.

[bib62] Reynolds D , Thomas T. Evolution and function of eukaryotic-like proteins from sponge symbionts. Mol Ecol. 2016;25:5242–53. 27543954 10.1111/mec.13812

[bib64] Roderer D , Hofnagel O, Benz R et al. Structure of a Tc holotoxin pore provides insights into the translocation mechanism. Proc Natl Acad Sci. 2019;116:23083–90. 31666324 10.1073/pnas.1909821116 PMC6859359

[bib63] Roderer D , Raunser S. Tc toxin complexes: assembly, membrane permeation, and protein translocation. Annu Rev Microbiol. 2019;73:247–65. 31140906 10.1146/annurev-micro-102215-095531

[bib65] Rojas MI , Cavalcanti GS, McNair K et al. A distinct contractile injection system gene cluster found in a majority of healthy adult human microbiomes. mSystems. 2020;5:e00648–20. 32723799 10.1128/mSystems.00648-20 PMC7394362

[bib66] Samba-Louaka A , Delafont V, Rodier MH et al. Free-living amoebae and squatters in the wild: ecological and molecular features. FEMS Microbiol Rev. 2019;43:415–34. 31049565 10.1093/femsre/fuz011

[bib67] Sanders JG , Beinart RA, Stewart FJ et al. Metatranscriptomics reveal differences in in situ energy and nitrogen metabolism among hydrothermal vent snail symbionts. ISME J. 2013;7:1556–67. 23619306 10.1038/ismej.2013.45 PMC3721115

[bib68] Santos-Garcia D , Rollat-Farnier PA, Beitia F et al. The genome of *Cardinium* cBtQ1 provides insights into genome reduction, symbiont motility, and its settlement in *Bemisia**tabaci*. Genome Biol Evolut. 2014;6:1013–30. 10.1093/gbe/evu077 PMC4007549 24723729

[bib69] Schloss PD , Westcott SL, Ryabin T et al. Introducing mothur: open-source, platform-independent, community-supported software for describing and comparing microbial communities. Appl Environ Microbiol. 2009;75:7537–41. 19801464 10.1128/AEM.01541-09 PMC2786419

[bib71] Schmitz-Esser S , Linka N, Collingro A et al. ATP/ADP translocases: a common feature of obligate intracellular amoebal symbionts related to Chlamydiae and Rickettsiae. J Bacteriol. 2004;186:683–91. 14729693 10.1128/JB.186.3.683-691.2004 PMC321502

[bib70] Schmitz-Esser S , Penz T, Spang A et al. A bacterial genome in transition - an exceptional enrichment of IS elements but lack of evidence for recent transposition in the symbiont *Amoebophilus asiaticus*. BMC Evol Biol. 2011;11:270. 21943072 10.1186/1471-2148-11-270 PMC3196728

[bib73] Schmitz-Esser S , Tischler P, Arnold R et al. The genome of the amoeba symbiont “Candidatus Amoebophilus asiaticus” reveals common mechanisms for host cell interaction among amoeba-associated bacteria. J Bacteriol. 2010;192:1045–57. 20023027 10.1128/JB.01379-09 PMC2812958

[bib72] Schmitz-Esser S , Toenshoff ER, Haider S et al. Diversity of bacterial endosymbionts of environmental acanthamoeba isolates. Appl Environ Microbiol. 2008;74:5822–31. 18641160 10.1128/AEM.01093-08 PMC2547052

[bib74] Shikuma NJ , Pilhofer M, Weiss GL et al. Marine tubeworm metamorphosis induced by arrays of bacterial phage tail-like structures. Science. 2014;343:529–33. 24407482 10.1126/science.1246794 PMC4949041

[bib75] Siguier P , Gourbeyre E, Chandler M. Bacterial insertion sequences: their genomic impact and diversity. FEMS Microbiol Rev. 2014;38:865–91. 24499397 10.1111/1574-6976.12067 PMC7190074

[bib77] Stones DH , Krachler AM. Against the tide: the role of bacterial adhesion in host colonization. Biochem Soc Trans. 2016;44:1571–80. 27913666 10.1042/BST20160186 PMC5134996

[bib76] Stones DH , Krachler AM. Fatal attraction: how bacterial adhesins affect host signaling and what we can learn from them. Int J Mol Sci. 2015;16:2626–40. 25625516 10.3390/ijms16022626 PMC4346855

[bib78] Sunagawa S , Woodley CM, Medina M. Threatened corals provide underexplored microbial habitats. PLoS ONE. 2010;5:e9554. 20221265 10.1371/journal.pone.0009554 PMC2832684

[bib79] Toft C , Andersson SGE. Evolutionary microbial genomics: insights into bacterial host adaptation. Nat Rev Genet. 2010;11:465–75. 20517341 10.1038/nrg2798

[bib80] Voth DE . ThANKs for the repeat: intracellular pathogens exploit a common eukaryotic domain. Cell Logistics. 2011;1:128–32. 10.4161/cl.1.4.18738 PMC3265924 22279611

[bib81] Wang Z , Wu M. Comparative genomic analysis of *Acanthamoeba* endosymbionts highlights the role of amoebae as a “melting pot” shaping the *Rickettsiales* evolution. Genome Biol Evolut. 2017;9:3214–24. 10.1093/gbe/evx246 PMC5751055 29177480

[bib82] Ward JH . Hierarchical grouping to optimize an objective function. J Am Statist Assoc. 1963;58:236–244.

[bib83] Warnes G , Bolker B, Bonebakker L et al. gplots: various r programming tools for plotting data. 2009. https://cran.r-project.org/web/packages/gplots/index.html.

[bib84] Westermann AJ , Gorski SA, Vogel J. Dual RNA-seq of pathogen and host. Nat Rev Microbiol. 2012;10:618–30. 22890146 10.1038/nrmicro2852

[bib85] Whitworth T , Popov VL, Yu XJ et al. Expression of the *Rickettsia**prowazekii pld* or *tlyC* gene in *Salmonella**enterica* serovar typhimurium mediates phagosomal escape. Infect Immun. 2005;73:6668–73. 16177343 10.1128/IAI.73.10.6668-6673.2005 PMC1230948

[bib86] Xuan YH , Yu HS, Jeong HJ et al. Molecular characterization of bacterial endosymbionts of *Acanthamoeba* isolates from infected corneas of Korean patients. Korean J Parasitol. 2007;45:1–9. 17374972 10.3347/kjp.2007.45.1.1 PMC2526339

[bib87] Zhou Y , Zhu Y. Diversity of bacterial manipulation of the host ubiquitin pathways. Cell Microbiol. 2015;17:26–34. 25339545 10.1111/cmi.12384

[bib88] Zinicola M , Higgins H, Lima S et al. Shotgun metagenomic sequencing reveals functional genes and microbiome associated with bovine digital dermatitis. PLoS ONE. 2015a;10:e0133674. 26193110 10.1371/journal.pone.0133674 PMC4508036

[bib89] Zinicola M , Lima F, Lima S et al. Altered microbiomes in bovine digital dermatitis lesions, and the gut as a pathogen reservoir. PLoS ONE. 2015b;10:e0120504. 25781328 10.1371/journal.pone.0120504 PMC4362943

