# Supplementary material for: The life cycle-dependent transcriptional profile of the obligate intracellular amoeba symbiont Amoebophilus asiaticus
Source: FEMS Microbiol Ecol. 2022 Jan 6;98(1):fiac001. doi: 10.1093/femsec/fiac001 (PMC8831229; doi:10.1093/femsec/fiac001)

Figure S1. Characteristic life cycle stages of *A. asiaticus* in its *Acanthamoeba* host. Representative Fluorescence *in situ* hybridization (FISH) images of *A. asiaticus* 5a2 inside its natural host amoebae at three different time points post infection (p. i.) are shown. Amoebae are shown in blue (Cy5-labelled probe EUK516), *A. asiaticus* 5a2 is shown in yellow (overlay of the Cy3-labelled *A. asiaticus* probe Aph1180 (red) and the Fluos-labelled general bacterial probe EUB338 (green)).

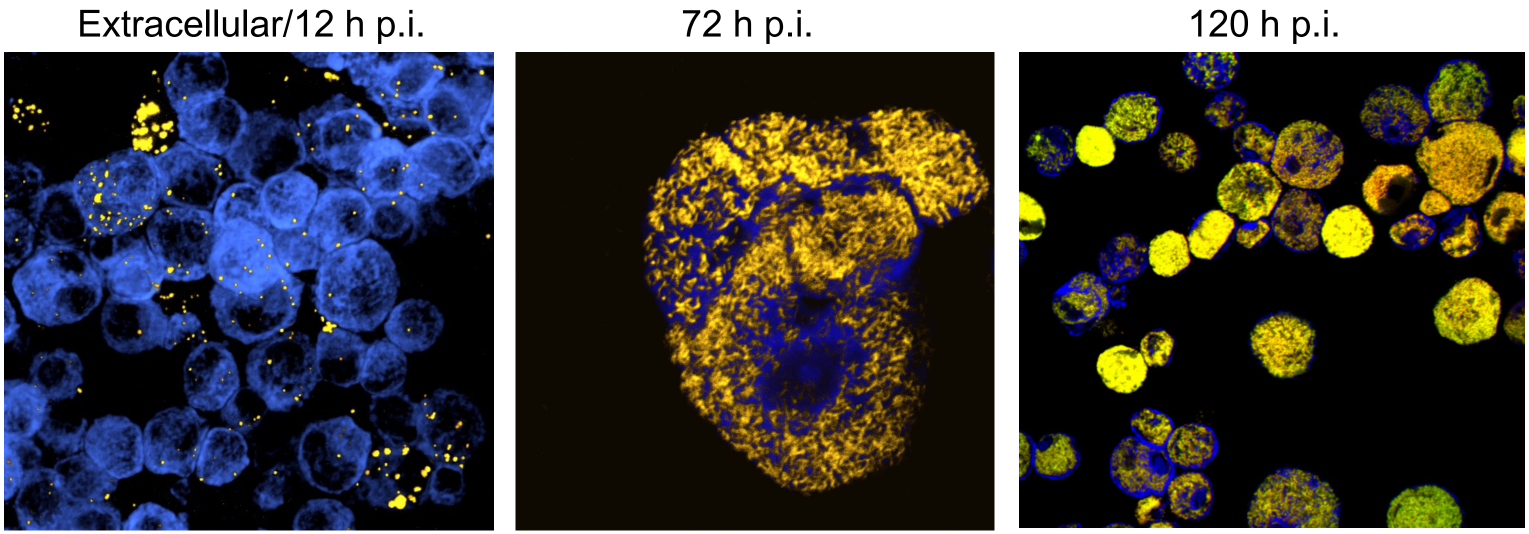

Supplement: fiac001_Supplemental_Files [file fiac001_supplemental_files.zip › Figure_S1-12-20-2021.pdf]
