# Supplementary material for: The life cycle-dependent transcriptional profile of the obligate intracellular amoeba symbiont Amoebophilus asiaticus
Source: FEMS Microbiol Ecol. 2022 Jan 6;98(1):fiac001. doi: 10.1093/femsec/fiac001 (PMC8831229; doi:10.1093/femsec/fiac001)

Figure S2. Variation in global gene expression at different time points of the *A. asiaticus* live cycle revealed by principal component analysis. The normalized differences in expression patterns were used for the distance matrix. EC= extracellular, 12= 12 h p.i., 72= 72 h p.i., 144= 144 h p.i.

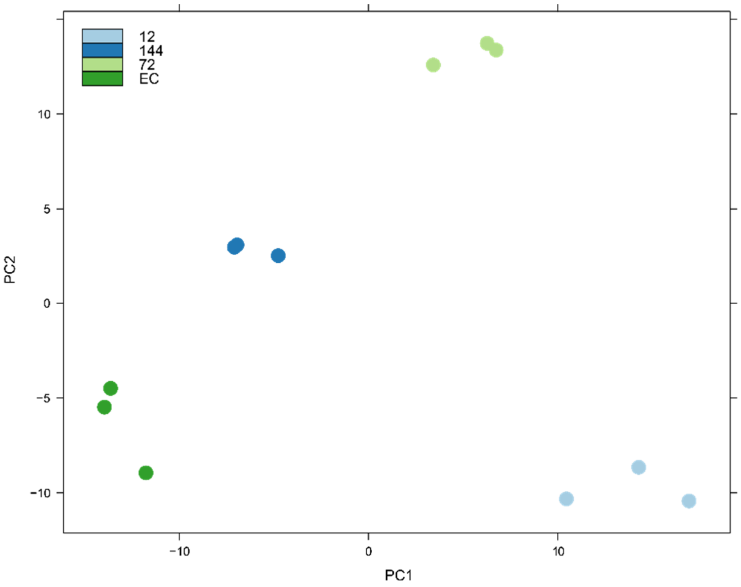

Supplement: fiac001_Supplemental_Files [file fiac001_supplemental_files.zip › Figure_S2-12-20-2021.pdf]
