# Supplementary material for: The life cycle-dependent transcriptional profile of the obligate intracellular amoeba symbiont Amoebophilus asiaticus
Source: FEMS Microbiol Ecol. 2022 Jan 6;98(1):fiac001. doi: 10.1093/femsec/fiac001 (PMC8831229; doi:10.1093/femsec/fiac001)

Figure S3. Hierarchical clustering of constitutively expressed genes (n=672). All genes not being differentially expressed were considered constitutively expressed. Four main clusters representing four different expression levels were defined based on the dendrogram's branching hierarchy. Green= low expression, yellow= moderate expression, orange= high expression, red= very high expression.

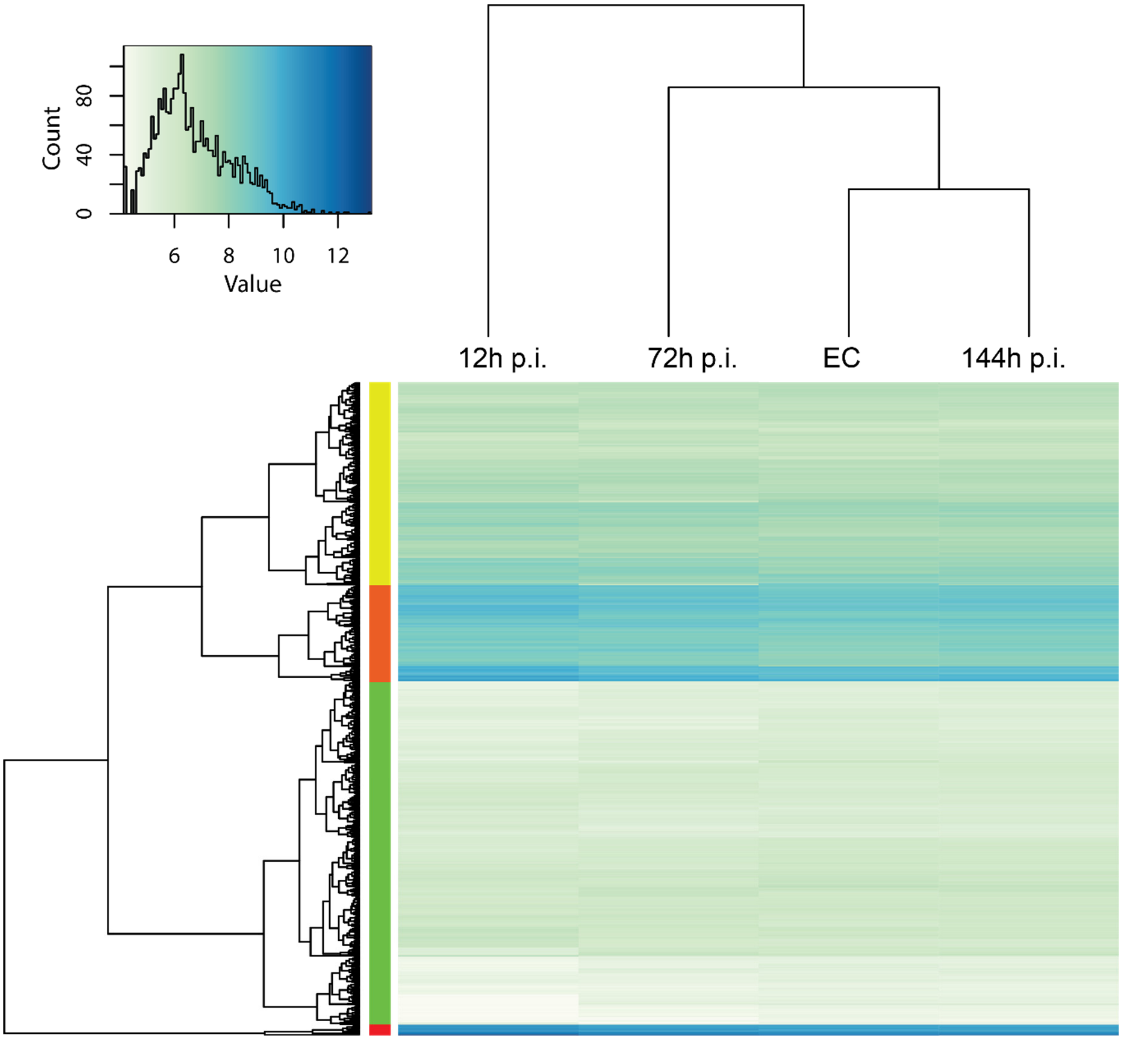

Supplement: fiac001_Supplemental_Files [file fiac001_supplemental_files.zip › Figure_S3-12-20-2021.pdf]
