# Supplementary material for: The life cycle-dependent transcriptional profile of the obligate intracellular amoeba symbiont Amoebophilus asiaticus
Source: FEMS Microbiol Ecol. 2022 Jan 6;98(1):fiac001. doi: 10.1093/femsec/fiac001 (PMC8831229; doi:10.1093/femsec/fiac001)

Figure S4. Hierarchical clustering of gene expression values of leucine-rich repeat harboring genes (n=20 genes). The locus_tags of all genes are listed. Dispersion estimates in DeSeq were done with the local fit type, as parametric dispersion fit failed.

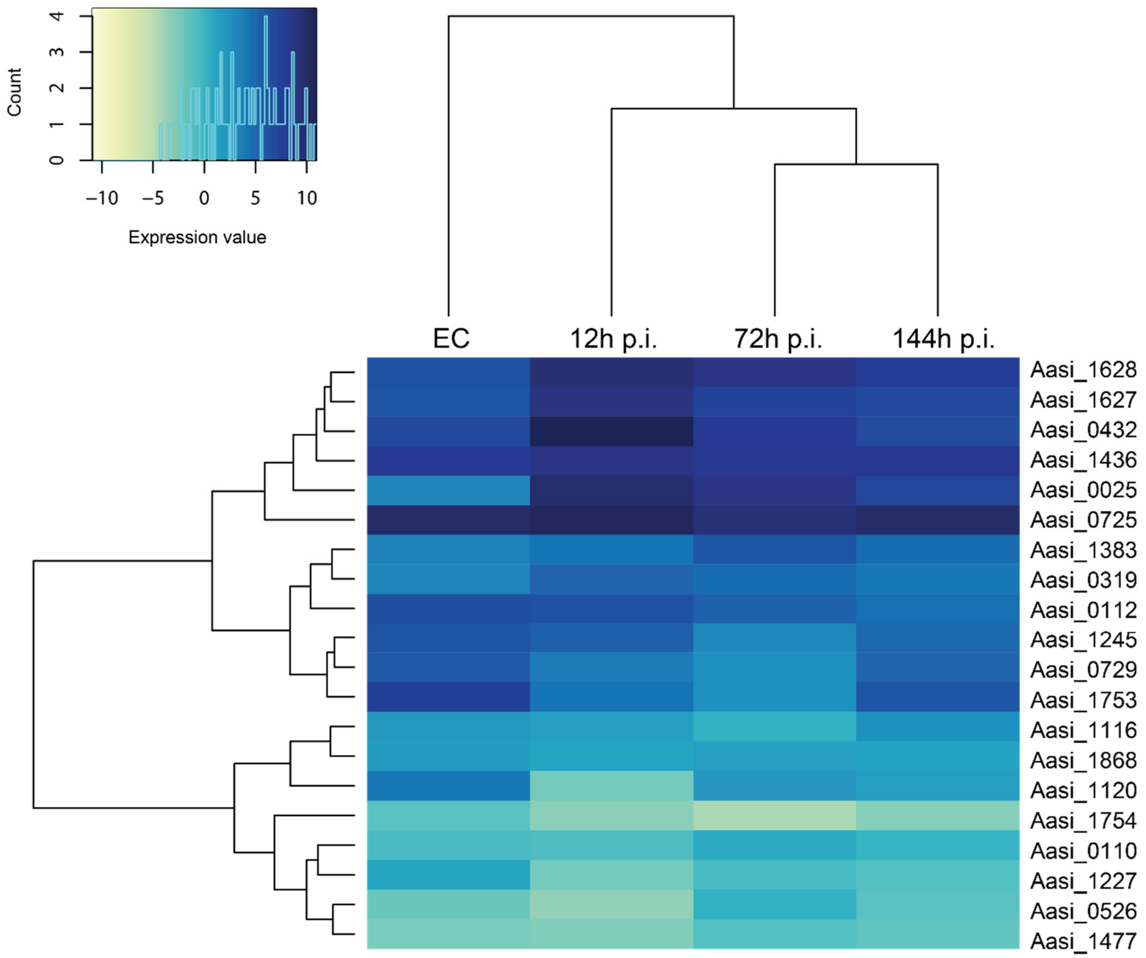

Supplement: fiac001_Supplemental_Files [file fiac001_supplemental_files.zip › Figure_S4-12-20-2021.pdf]
