# Supplementary material for: The life cycle-dependent transcriptional profile of the obligate intracellular amoeba symbiont Amoebophilus asiaticus
Source: FEMS Microbiol Ecol. 2022 Jan 6;98(1):fiac001. doi: 10.1093/femsec/fiac001 (PMC8831229; doi:10.1093/femsec/fiac001)

Figure S6. Hierarchical clustering of gene expression values of ANK repeat harboring genes (n=51 genes). The locus_tags of highly expressed ANK repeat genes are listed.

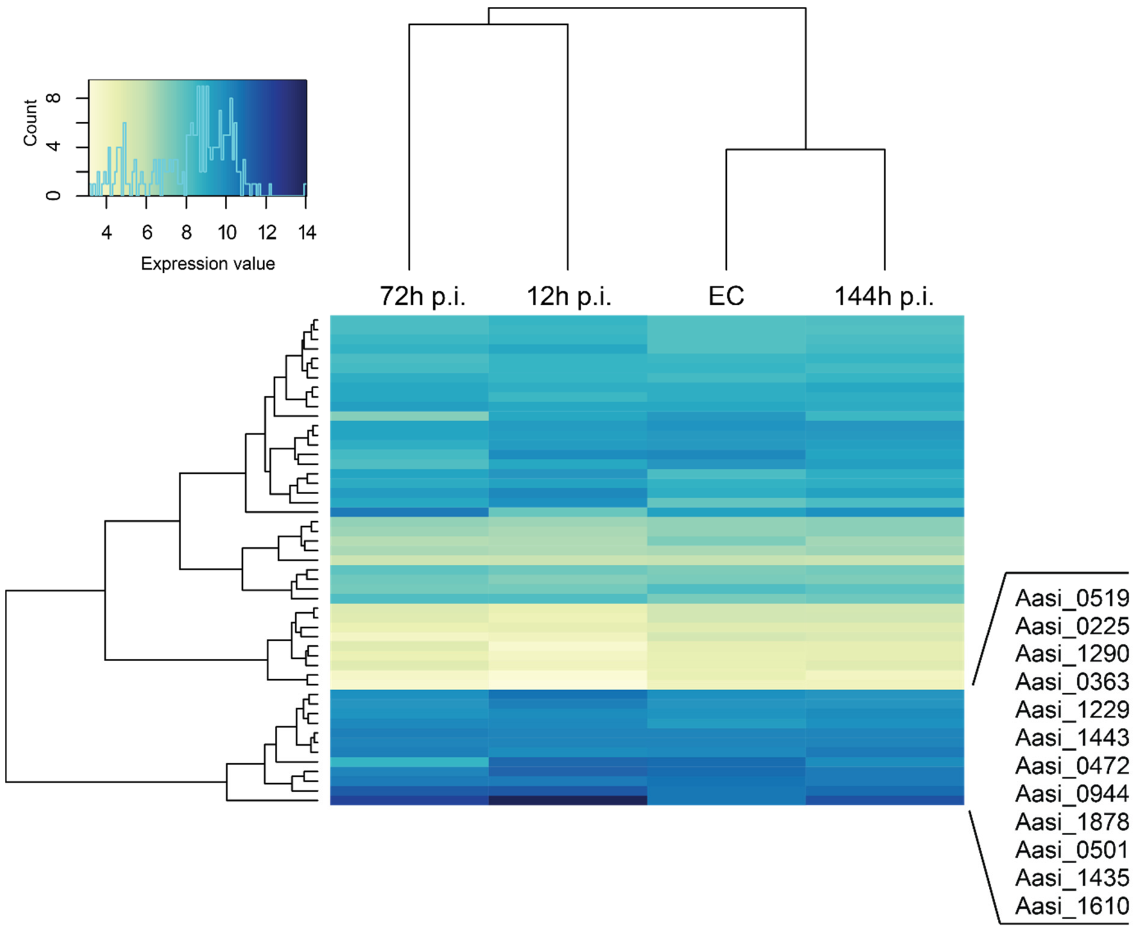

Supplement: fiac001_Supplemental_Files [file fiac001_supplemental_files.zip › Figure_S6-12-20-2021.pdf]
