# Supplementary material for: The life cycle-dependent transcriptional profile of the obligate intracellular amoeba symbiont Amoebophilus asiaticus
Source: FEMS Microbiol Ecol. 2022 Jan 6;98(1):fiac001. doi: 10.1093/femsec/fiac001 (PMC8831229; doi:10.1093/femsec/fiac001)
Supplement: fiac001_Supplemental_Files [file fiac001_supplemental_files.zip › Table S1 10-29-2021.pdf]

**Table S1. RNA-seq read statistics: The number of processed and assigned reads is shown.** The single best match coverage represents the percentage of the reference sequence that is covered by at least one read of the single best match mapping class (Hilker et al., 2014). EC= extracellular stage, h p.i.= hours post infection.

| Dataset statistics | **EC** | | | **12 h p.i.** | | | **72 h p.i.** | | | **144 h p.i.** | | |
|---|---|---|---|---|---|---|---|---|---|---|---|---|
| | 1st replicate | 2nd replicate | 3rd replicate | 1st replicate | 2nd replicate | 3rd replicate | 1st replicate | 2nd replicate | 3rd replicate | 1st replicate | 2nd replicate | 3rd replicate |
| Total number of reads | 245,849,405 | 230,843,854 | 249,002,487 | 235,660,189 | 236,209,409 | 238,550,608 | 240,577,273 | 228,586,894 | 243,128,229 | 238,633,539 | 243,599,851 | 224,565,686 |
| Trimmed reads | 245,396,423 | 230,180,353 | 228,586,894 | 200,391,989 | 235,649,861 | 238,027,804 | 239,451,629 | 228,073,810 | 240,331,445 | 214,056,768 | 228,586,894 | 222,427,262 |
| Trimmed unique reads | 7,658,964 | 8,344,474 | 10,699,707 | 13,256,504 | 16,636,556 | 15,238,768 | 17,580,679 | 11,986,307 | 10,813,003 | 13,490,823 | 12,292,695 | 20,024,472 |
| Trimmed unique reads assigned to the *A. asiaticus* 5a2 genome (percentage) | 2,075,515 (27%) | 3,160,565 (38%) | 2,100,648 (20%) | 280,860 (2%) | 246,985 (1%) | 187,983 (1%) | 101,7541 (6%) | 682,467 (6%) | 499,366 (5%) | 3,137,270 (23%) | 2,760,644 (22%) | 2,643,681 (13%) |
| Theoretical redundancy of coverage | 55.1× | 83.8× | 55.7× | 7.5× | 6.6× | 5× | 27× | 18.1× | 13.3× | 83.2× | 73.3× | 70.1× |
| Single best match coverage | 98.7% | 99.6% | 99.1% | 76.2% | 70.2% | 64.5% | 96.4% | 93.8% | 91.7% | 99.8% | 99.5% | 99.5% |
