# Supplementary material for: The life cycle-dependent transcriptional profile of the obligate intracellular amoeba symbiont Amoebophilus asiaticus
Source: FEMS Microbiol Ecol. 2022 Jan 6;98(1):fiac001. doi: 10.1093/femsec/fiac001 (PMC8831229; doi:10.1093/femsec/fiac001)
Supplement: fiac001_Supplemental_Files [file fiac001_supplemental_files.zip › Table S4 10-29-2021.pdf]

Table S4. The number of genes up- and downregulated during the life cycle of *A. asiaticus* are listed. 884 genes were significantly up- or downregulated at least once during the life cycle. EC= extracellular stage, h p.i. = hours post infection.

	**Number of genes upregulated**	**Number of genes downregulated**
**12 h p.i.-72 h p.i.**	102	205
**72 h p.i.-144 h p.i.**	267	239
**144 h p.i.-EC**	250	231
**EC-12 h p.i.**	274	199
